# Genome-Wide Association Studies of Maize Seedling Root Traits under Different Nitrogen Levels

**DOI:** 10.3390/plants11111417

**Published:** 2022-05-26

**Authors:** Yafang Fu, Jianchao Liu, Zhenqing Xia, Qi Wang, Shibo Zhang, Guixin Zhang, Haidong Lu

**Affiliations:** College of Agronomy, Northwest A&F University, Yangling, Xianyang 712100, China; f13546632915@163.com (Y.F.); ljcnwsuaf@nwsuaf.edu.cn (J.L.); 17854233612@163.com (Z.X.); qiwangnwafu@163.com (Q.W.); z2335570357@163.com (S.Z.); zgx199869@163.com (G.Z.)

**Keywords:** genome-wide association study (GWAS), nitrogen, maize, root, candidate genes

## Abstract

Nitrogen (N) is one of the important factors affecting maize root morphological construction and growth development. An association panel of 124 maize inbred lines was evaluated for root and shoot growth at seedling stage under normal N (CK) and low N (LN) treatments, using the paper culture method. Twenty traits were measured, including three shoot traits and seventeen root traits, a genome-wide association study (GWAS) was performed using the Bayesian-information and Linkage-disequilibrium Iteratively Nested Keyway (BLINK) methods. The results showed that LN condition promoted the growth of the maize roots, and normal N promoted the growth of the shoots. A total of 185 significant SNPs were identified, including 27 SNPs for shoot traits and 158 SNPs for root traits. Four important candidate genes were identified. Under LN conditions, the candidate gene *Zm00001d004123* was significantly correlated with the number of crown roots, *Zm00001d025554* was correlated with plant height. Under CK conditions, the candidate gene *Zm00001d051083* was correlated with the length and area of seminal roots, *Zm00001d050798* was correlated with the total root length. The four candidate genes all responded to the LN treatment. The research results provide genetic resources for the genetic improvement of maize root traits.

## 1. Introduction

Nitrogen (N) metabolism is one of the most basic metabolic processes in plants. For maize, N is the most absorbed mineral element in its life. However, when the amount of N fertilizer exceeds the seasonal requirement or the application method is inappropriate, N is easily lost through rainwater washing, runoff, volatilization and other ways, causing pollution to the surrounding atmosphere and the water environment (Yu et al., 2021) [1]. The N use efficiency (NUE) in China is generally between 30% and 35%, much lower than that in agriculture-developed countries, where the NUE is as high as 70% (Liu et al., 2010) [2]. Therefore, it is very important to cultivate N-efficient maize varieties for maintaining high maize yield and protecting the ecological environment in China.

The maize root system is composed of a variety of complex quantitative traits, with obvious gene interactions and gene-environment interactions (He, 2018) [3]. Favorable root morphology is beneficial for maize to efficiently acquire N from the soil, and an appropriate supply of N is conducive to the growth and development of the maize root system (Yu et al., 2015) [4]. Appropriate application of N can promote root growth, increase the number of root hairs, and increase the total root surface area, which is conducive to expanding the root area and increasing the root depth (Zhang, 2017) [5]. Low N conditions are conducive to the vertical growth of roots, with a significant increase in total root length (Wang et al., 2002) [6]. The root biomass of the maize genotype with high N efficiency was significantly higher than that of the maize genotype with low N efficiency under low N conditions, but the difference was very small under high N conditions (Wang et al., 2002) [6]. Breeding maize varieties with N efficient root architecture is beneficial to improve NUE and reduce the application of N fertilizer.

Up to now, there have been many reports on the genes of root traits and NUE in maize. Wang et al. (2019) [7] studied the natural variation of thirteen root traits and three shoot traits in two-hundred and ninety-seven maize inbred lines. A total of five candidate genes significantly associated with the roots were detected. Among them, *GRMZM2G138338* may encode an inactive leucine-rich repeat receptor-like protein kinase, which was significantly related to root length. He et al. (2020) [8] conducted the N efficiency-related traits of 139 maize inbred lines under two N levels and identified that the *GRMZM2G175140* and *GRMZM2G108597-*encoded ammonium transporter1 and transmembrane amino acid transporter family protein, respectively, may be important candidate genes for NUE. Sun et al. (2020) [9] used 461 maize inbred lines; GWAS was performed on root traits at the seedling stage under two N levels. Under LN conditions, the protoporphyrinogen IX oxidase 2 gene *GRMZM2G364901* was associated with the total root surface area and the DELLA protein-encoding gene *GRMZM2G144744* was associated with the length of the visible lateral root zone of the primary root. Morosini et al. (2017) [10] used 64 tropical maize inbred lines as materials and detected seven maize loci significantly associated with low nitrogen tolerance and root traits. The predicted candidate genes *GRMZM2G131340* and *GRMZM2G306935* were involved mostly in transcriptional regulation and enzyme activity in the N cycle.

Although many maize root and nitrogen efficiency genes have been discovered, few have been actually cloned and applied. However, in model crops such as rice, there was already plenty of precedent. Liu et al. (2021) [11] used 110 rice germplasm materials for N response assessment; the results showed that the response of tiller N was highly correlated with the variation in the N use efficiency of the rice population. Using GWAS, A candidate gene locus, *OsTCP19*, highly correlated with the tillering N response, was identified. Through genetic analysis, the transcriptional expression of *OsTCP19* itself is negatively regulated by the external N levels. At the same time, *OsTCP19* directly inhibits tiller and promotes the expression of gene DLT, so it has the function of regulating the response of the tiller. N. Lou et al. (2021) [12] conducted GWAS on the endosperm of 533 rice varieties, a total of 343 significant loci were detected on 12 chromosomes and performed a functional analysis of one of the candidate genes (*LOC_Os03g48060*; named *FLO19* in the study). The candidate gene *FLO19* encodes the class I transglutaminase. The knock-out mutant *FLO19* was obtained, and the hydroponic experiment was carried out with the wild variety TH11. The results showed that, compared with the wild varieties, *FLO19* mutants had impaired carbon and N metabolism, decreased plant height, increased root length and decreased grain quality. These successful examples using rice pointed the way for our research.

In this study, 124 maize inbred lines were selected to form a genetic population, 20 N efficiency-related traits were surveyed to carry out GWAS by using the BLINK method in maize at the seedling stage. Our objectives were: (1) Mining the key genetic sites and candidate genes of related traits under low N stress; (2) Explaining the relationship between phenotypic changes and genetic polymorphisms in the maize seedling stage; Providing a genetic basis for the improvement of N-efficient maize varieties.

## 2. Materials and Methods

### 2.1. Plant Materials

An association panel of 124 maize inbred lines was used in this study, of which 97 inbred lines were elite Chinese inbred lines, and the other 27 were newly bred germplasms selected from the Shaan A group and Shaan B group, two maize heterosis groups selected by the research group in recent years. In the process of construction, the breeding ideas of multi-location, high density, low N and drought were followed, which greatly improves the stress resistance of inbred lines (Li et al., 2018; Zhao et al., 2019; He et al., 2020) [8,13,14]. From 2007 to 2008, basic groups were constructed over three generations. From 2009 to 2015, the Shaan A group and Shaan B group were optimized and upgraded through seven rounds of selection in thirty departments in seven provinces (Shaanxi, Gansu, Henan, Hebei, Neimenggu, Sichuan and Xinjiang). The detailed inbred line materials are shown in Table S6, Supplementary Materials.

### 2.2. Experimental Design

In this study, the paper rolls’ culture method was used to cultivate and process the research materials. The experiment set two treatments with CK and LN, and two independent experiments were carried out. The method selected the full and uniform size of maize seeds, soaked them in 10% H_2_O_2_ for 20 min, washed them with distilled water, and soaked them in saturated CaSO_4_ for 6 h to disinfect the seeds. The brown seed germination papers (10 in × 15 in) from the Anchor Company of the United States were pre-wetted with 2.5 g/L fungicide solution, Captan, to eliminate the possibility of fungal growth during seedling development. Then, the germinated papers with eight seeds were placed vertically in 2000 mL large beakers containing autoclaved standard Hoagland nutrient solution. The nutrient solution in the beakers was replaced every 2 days and positions were changed frequently. The growth conditions of the seedlings were: photoperiod 8/16 (day/night); day/night temperature 22 °C/25 °C; light intensity of 200 μmol·m^−2^·s^−1^; relative humidity of 65–75%. Hoagland nutrient solution in CK consisted of (mmol·L^−1^): Ca(NO_3_)_2_ 2.0, K_2_SO_4_ 0.75, MgSO_4_ 0.65, KCl 0.1, KH_2_PO_4_ 0.25, H_3_BO_3_ 1 × 10^−3^, MnSO_4_ 1 × 10^−3^, CuSO_4_ 1 × 10^−4^, ZnSO_4_ 1 × 10^−3^, (NH_4_)_6_Mo_7_O_24_ 5 × 10^−6^, Fe-EDTA 0.1. For LN treatment, no Ca (NO3)_2_ was supplied. The Ca^2^^+^ was complemented by adding CaCl_2_ to the same level, as in the CK treatment. The pH of the nutrient solution was adjusted to 6.0 with NaOH.

Plants were harvested when the maize seedlings reached the three-leaf stage (about 14 days after germination); three healthy seedlings in each paper roll were selected for trait determination. The plants were divided into shoots and roots from the first whorl crown roots, and the seeds were removed from the roots. Phenotypic observations are presented in Figure S7, Supplementary Materials.

### 2.3. Root and Shoot Phenotype Measurement

A total of 20 seedling phenotypic traits were measured, including 3 shoot traits and 17 root traits. For the shoot traits, the chlorophyll content of leaves, as represented by the SPAD value, and the SPAD value of the second leaf was measured with chlorophyll meter model SPAD-502. The plant height (PH) was measured using a ruler. After 70 °C drying in an oven, the shoot dry weight (SDW) was weighed on a balance with an accuracy of 0.001. The roots were stored at −20 °C until measured. The primary root length (PRL), the seminal root length (SRL) and the crown root length (CRL) were measured with a ruler. The seminal root number (SRN)and the crown root number (CRN) were measured by counting. The root system was divided into four parts: primary root; seminal root; crown root and lateral root, and samples of each part were floated in water in a transparent plastic tray and scanned with a scanner. The traits of the crown root surface area (CRSA), crown root volume (CRV), seminal root surface area (SRSA), seminal root volume (SRV), primary root surface area (PRSA), primary root volume (PRV), total root length (TRL), total root surface area (TRSA), total root volume (TRV) and total lateral root length (TLRL) were scanned and analyzed using the WinRHIZO 2004b software. The root dry weight was also dried at 70 °C until of a constant weight and was weighed on a balance with an accuracy of 0.001. Then the root-to-shoot ratio (RSR) was calculated. The traits are described in Table 1.

### 2.4. Phenotypic Data Analysis

The statistical analysis software IBM SPSS Statistics 26 was used to carry out descriptive statistical analysis, analysis of variance and correlation analysis of the phenotypic data. The original 2018 software was used to draw the graphics. The generalized heritability calculation formula (Nyquist and Baker, 1991) is h^2^ = σ^2^_G_/(σ^2^_G_ + σ^2^_GE_/n + σ^2^_e_/nr) × 100%. In the formula, h^2^ represents the generalized heritability; σ^2^_G_ represents the variance of the genotype; σ^2^_GE_ represents the variance of the interaction between the genotype and the environment; σ^2^_e_ represents the error term; r represents the number of repetitions, which is three in this experiment; *n* represents the number of environments, which in this experiment is two. The “lme4” in the R software was used to calculate the best linear unbiased prediction (BLUP) value for each trait, which was used for subsequent GWAS.

### 2.5. Genome-Wide Association Studies

Plink software was used to filter the genotype data to remove the polymorphic loci with the minimum allele frequency less than 0.05, and then used the “LD KNNi Imputation” to fill in the missing genotype data, and a total of 55805 SNPs was used for subsequent analysis. The Tassel 5.2 software (Buckler Laboratory, Cornell University) was used to analyze the linkage disequilibrium (LD) between a single chromosome of maize and the paired polymorphic site markers in the entire genome (Bradbury et al., 2007) [15]. In order to reduce the false positives when identifying significant association sites, the structure software was used to calculate the population structure (Q), the “GAPIT” software package of the R software was used to calculate the kinship (Tang et al., 2016) [16]. GWAS was performed using the Bayesian-information and Linkage-disequilibrium Iteratively Nested Keyway (BLINK) method in R. The experiment used the Bonferroni correction method to correct the *p* value after multiple hypothesis testing for GWAS to reduce the probability of false positives. *p* < 1/n was selected, the threshold was set as *p* < 1.8 × 10^−5^. (He et al., 2020; Li et al., 2013) [8,17].

### 2.6. Candidate Gene Analysis

The genome sequence of the maize line B73(RefGen_v4) (https://www.maizegdb.org/, (accessed on 20 May 2021) in the Maize GDB was used as the reference for the candidate gene prediction. The confidence interval of significant SNPs was determined, based on LD decayed at r^2^ = 0.1. The confidence interval of each significant SNP was used to determine the search scope of candidate genes. Preferentially select genes related to the N utilization process were obtained as the candidate genes. The candidate genes obtained from the association analysis were analyzed for GO enrichment through the website http://bioinfo.cau.edu.cn/agriGO, (accessed on 26 May 2021), and the significant GO term of the candidate genes was visualized. The most likely candidate genes were screened out, according to the function annotation of genes and their expression of maize seedlings.

### 2.7. Candidate Gene Expression Analysis

Combining the significant locus associated with the multiple traits and the BLUP value of the phenotypic traits of the associated population of two N environments, haplotype analysis was performed on the tested inbred lines. According to the results of the haplotype analysis, four inbred lines of different haplotypes were selected. Among them, 135 and 165 were the favorable haplotypes, and 116 and 160 were the unfavorable haplotypes. Using the culture method of 2.2, the root system samples of the three-leaf stage corn were obtained.

1. RNA extraction and cDNA preparation

The three-leaf stage hydroponic seedlings were taken as samples, and the total RNA was extracted after grinding with liquid nitrogen. The extraction steps refer to the TRNzol total RNA extraction instructions (AG). The integrity of the RNA was performed by agarose gel electrophoresis and stored at −80 °C for later use. The extracted RNA samples were reverse transcribed with the Fastking cDNA first-strand synthesis kit, and the cDNA template samples were detected with the primer ZmActin F/R of the maize internal reference gene and stored at −20 °C for later use.

2. Real-time fluorescent quantitative PCR

The spare reverse transcription product diluted to 10ng/ul was used as a cDNA template, ZmActin F/R was used as the internal standard primer, RT-qPCR analysis was performed using specific primers. Q7 was used as a RT-qPCR device, and the kit used was Super Real Premix Plus (SYBR Green). The reaction system and primers were shown in Table S5, Supplementary Materials. Three biological replicates were set up. Data were analyzed using the 2^-ΔΔCt^ method, based on the Ct values of the samples.

## 3. Results

### 3.1. Phenotypic Analysis of Shoot and Root Traits

**Figure 1:**
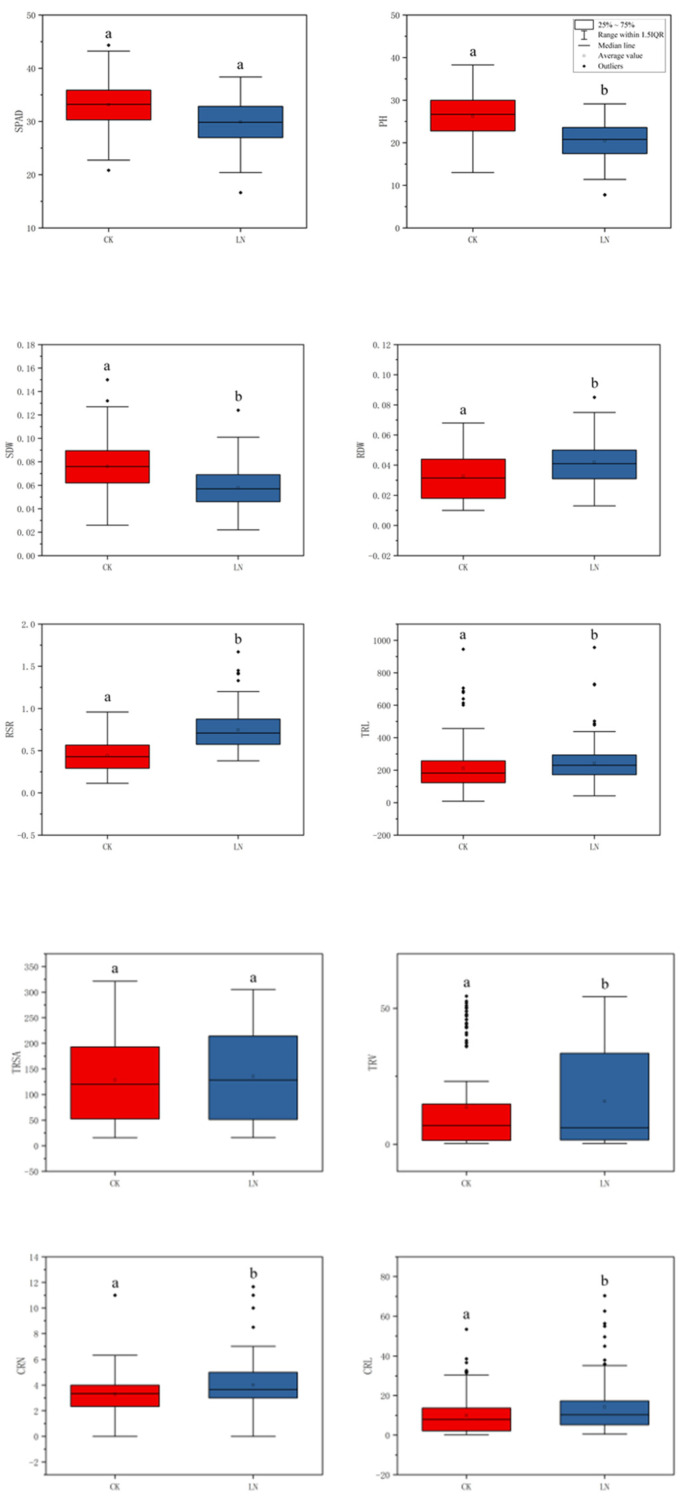

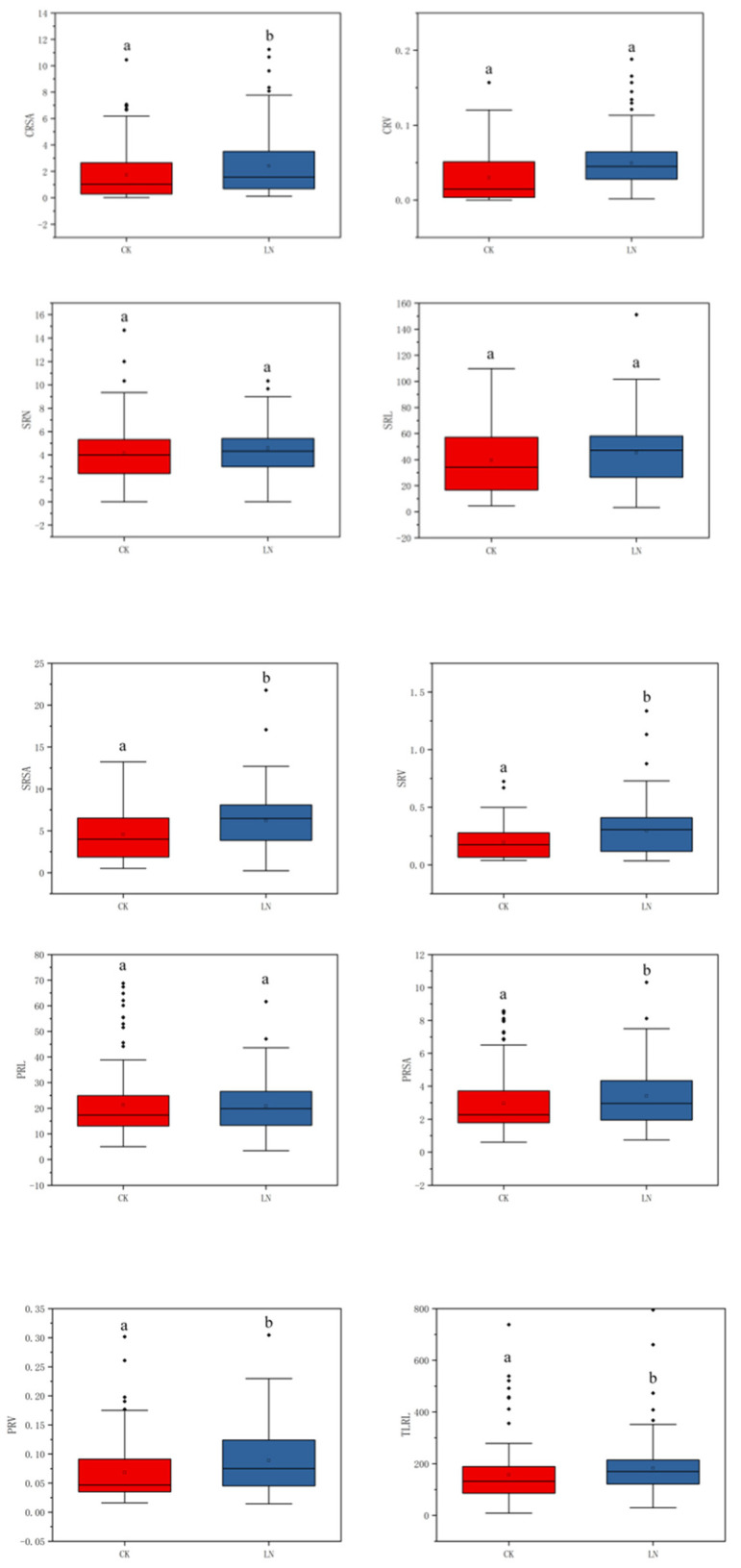
Phenotype data box plot for different nitrogen environments. In that order: SPAD value of the second leaf; Plant height; Shoot dry weight; Root dry weight; Root-to-shoot ratio; Total root length; Total root surface area; Total root volume; Crown root number; Crown root length; Crown root surface area; Crown root volume; Seminal root number; Seminal root length; Seminal root surface area; Seminal root volume; Primary root length; Primary root surface area; Primary root volume; Total lateral root length. In the Figure, the upper edge of the boxplot represents the maximum value; the lower edge represents the minimum value; the open circles represent the mean; the filled circles represent outliers and contains a line representing the median. a and b indicate the significance between CK and LN (*p* < 0.05). Red represents CK, blue represents LN.

In the association population, all of the traits have extensive variation at the CK and LN conditions, and they show obvious quantitative traits’ inheritance rules. Combining phenotypic data and boxplot analysis, the results show that there were differences between most traits at the two N levels, Only TRSA, SRN and PRL were not significantly different (Figure 1; Table S1, Supplementary Materials), indicating that TRSA, SRN and PRL were not easily affected by the N levels. Under the condition of CK, the shoot traits, SPAD, PH and SDW were higher than the LN level. While for the root traits, the results were opposite. (Figure 1; Table S1, Supplementary Materials). The results showed that under the conditions of sufficient N, the growth of new shoots was favorable, while under the condition of LN, the growth of the root system could be promoted.

**Figure 2:**
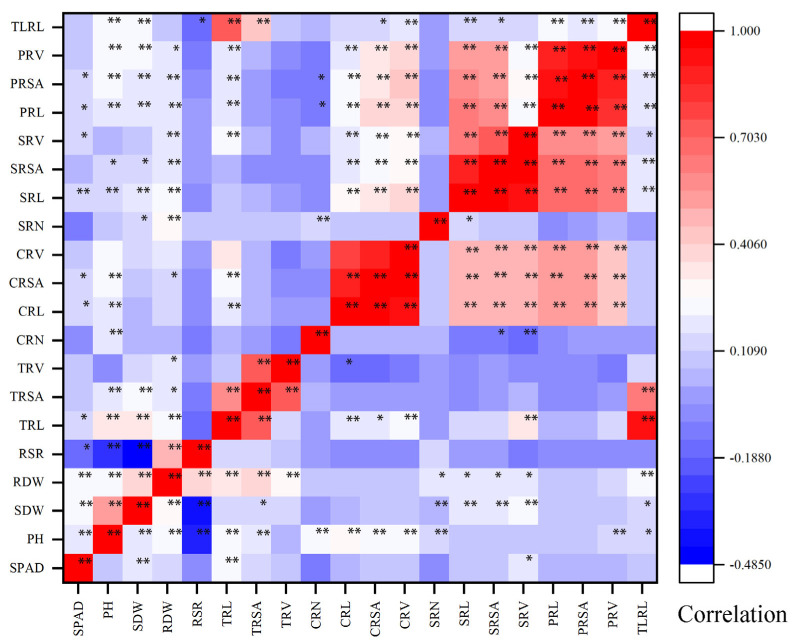
Phenotypic correlation analysis of different nitrogen levels. The upper left is the correlation of the CK treatment, and the lower right is the correlation of the LN treatment. Red represents positive correlation, blue represents negative correlation, and the darker the color, the greater the correlation. * and ** indicate significance at *p* < 0.05 and *p* < 0.01 between different traits.

RSR was significantly negatively correlated with PH and SDW, and significantly positively correlated with RDW at the two N levels. There was a significant negative correlation between RSR and SPAD under LN conditions, but there was no significant correlation under CK conditions., There was a significant positive correlation between the number of crown roots and the seminal roots under LN conditions, but no significant correlation under CK conditions. Except for CRN and SRN, there was a significant correlation between the other root traits at the two N levels (Figure 2). It showed that the root traits had a similar physiological basis, while a similar physiological basis exists between the shoot traits, and the relationship between the shoot and root systems was greatly affected by the environment. The results of the generalized heritability analysis showed that the heritability of these 20 traits ranged from 24.14% to 77.69%. (Table S1, Supplementary Materials). Among them, the minimum h^2^ was the generalized heritability of TRV in CK treatment (21.18%), and the largest h^2^ was the PH in the CK treatment (77.69%). Under the CK treatment, the heritability of PH and SDW was much greater than that of the LN treatment, and the heritability of CRN was much smaller than that of the LN treatment.

### 3.2. Population Structure and Linkage Disequilibrium Decay

**Figure 3:**
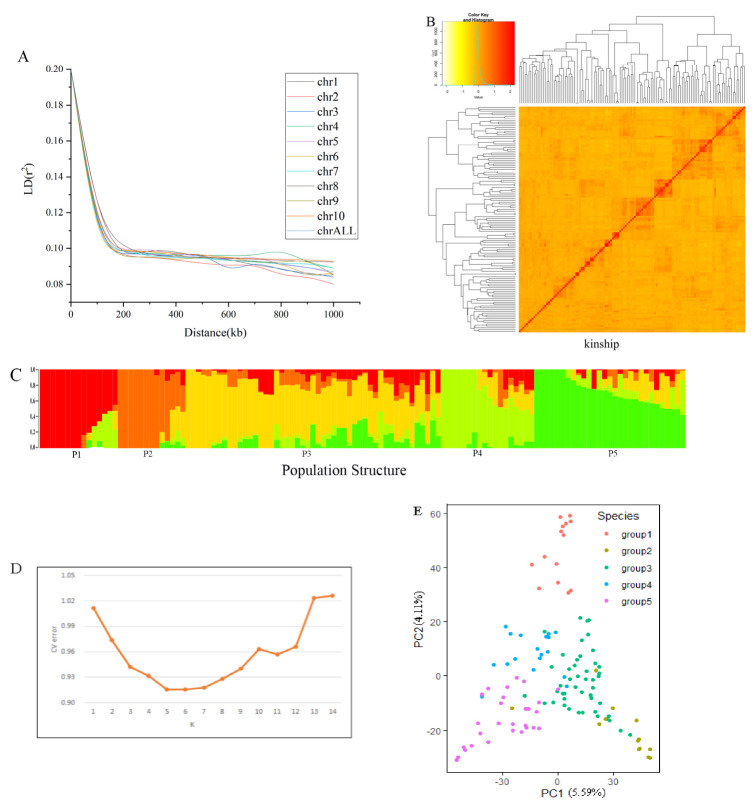
Group structure and genetic relationship analysis of related groups. (**A**). Linkage disequilibrium decay diagram. LD refers to the non-random association of alleles at different loci in a population; (**B**). Relationship analysis of association population; (**C**). Structure analysis results of association population. P1-P5 represent five distinct subpopulations. Different colors represent different ancestral subgroups. There are different colors in a bar, indicating that this inbred line is a cross between multiple ancestral subgroups; (**D**). Structure analysis of related populations. CV refers to the error value of cross-validation. The K value is the estimated number of distinct ancestral groups. The smaller the CV value, the more reliable the K value; (**E**). Principal component analysis. PC1 and PC2 were the two principal components that could explain most of the variation.

Structure software was used to analyze the structure of the association population; it was found that when the cross-validation error value was 5, the K value was the smallest (Figure 3D). The association population was divided into five subpopulations based on the population structure analysis and the PCA analysis (Figure 3 C,D). Each subpopulation contains 15, 13, 49, 18 and 29 accessions. It was found that there was no load group structure in this association population, which was suitable for subsequent association analysis. The results of the relative kinship analysis showed that the genetic relationship coefficient among most of the tested maize inbred lines was less than 0.1 (Figure 3B), indicating that the genetic relationship between individuals in the population was weak. With the increase of physical distance, the LD between the pairs of SNP markers showed a significant decreasing trend. The genome-wide LD analysis results showed that when r^2^ = 0.1, the LD decay distance was about 200 kb. (Figure 3A).

### 3.3. Genome-Wide Association Studies

Under the condition of controlling the population structure and kinship, the “GAPIT” of the R software was used to perform GWAS on shoot and root traits under two N conditions. A total of 185 significant associated SNPs were detected, 84 SNPs were detected under LN conditions, 101 SNPs were detected under CK conditions, of which 27 SNPs were shoot traits and 158 SNPs were root traits (Table 2 and Figure S1, Supplementary Materials). These SNPs were distributed on 10 chromosomes, among which the first and sixth chromosomes were more distributed, with 28 and 27 SNPs sites, respectively (Table S3, Supplementary Materials). Using the associated SNPs and LD decay distance, a total of 180 genes were identified as candidate genes based on the Zm-B73-4.0 reference genome annotation information (Table S3, Supplementary Materials).

**Figure 4:**
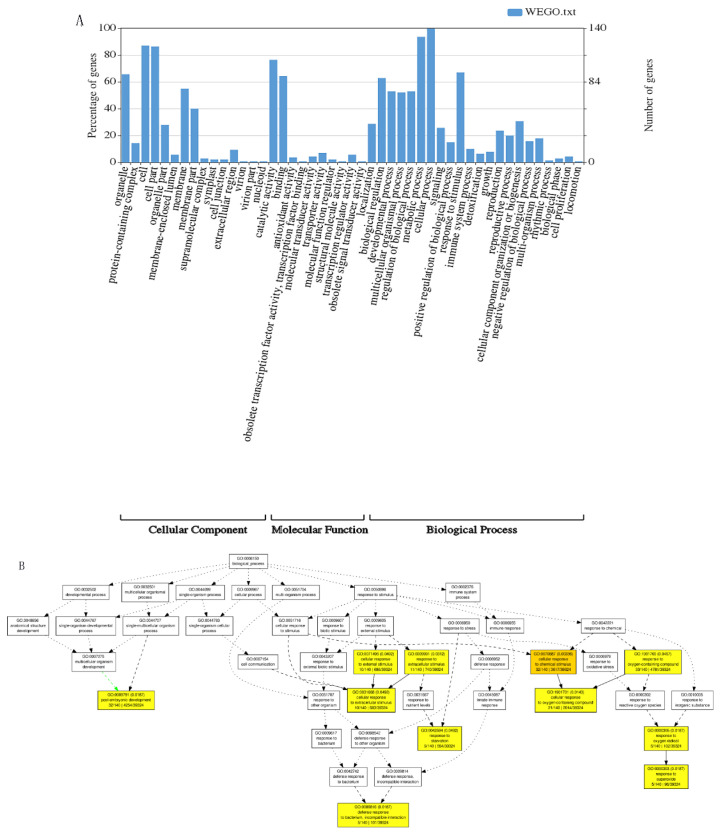
GO (Gene Ontology) terms related to nitrogen metabolism in candidate genes. (**A**). Histogram of GO enrichment analysis results; (**B**). DAG chart reflecting the upper and lower levels of GO term and the degree of enrichment.

The candidate genes detected by the GWAS analysis were subjected to enrichment analysis using the GO analysis method. The results were divided into three categories: cell components; molecular functions and biological processes. Cell components were mainly related to organelles, cell membranes and macromolecular complexes. Molecular functions were mainly manifested in the activity of catalysts, binders, regulators and transmembrane transport activities. The biological processes involved mainly included cell regulation, growth, immune, metabolic, negative regulation and response to stimuli, etc. (Figure 4A). A total of 10 significant GO terms were detected by the GO analysis of candidate genes. These GO terms were mainly related to the post-embryonic development of maize and the cellular response to external stimuli. These external factors mainly include oxygenated compounds, bacteria and nutrients (Figure 4B).

### 3.4. Tentative Candidate Genes for the Identified SNPs

**Figure 5:**
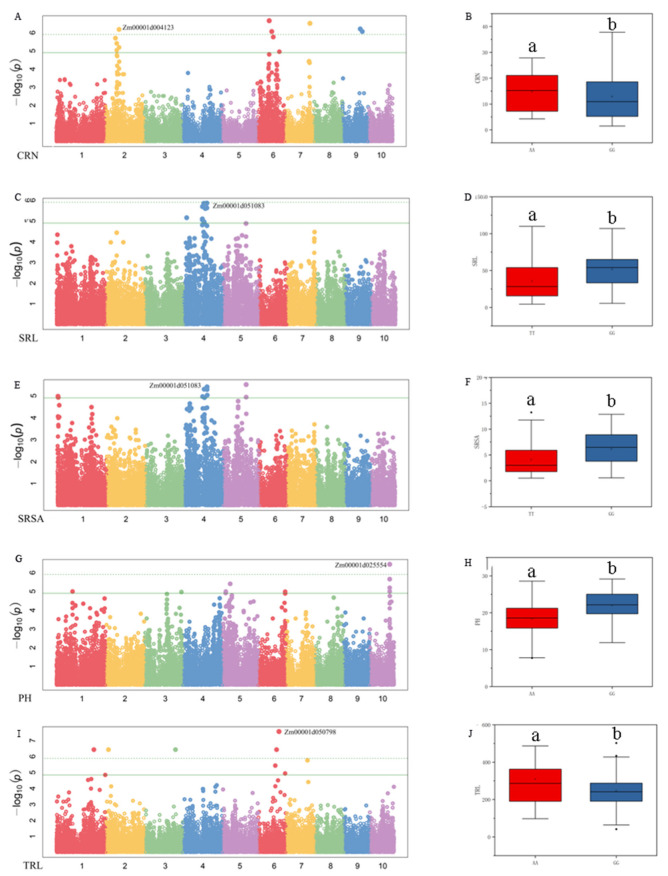
Manhattan plot of nitrogen efficiency-related traits and allelic effects of significant SNPs corresponding to genes. (**A**,**B**) Manhattan plot of CRN under LN and the *Zm00001d004123* corresponding to the allelic effect of significant SNP; (**C**,**D**) Manhattan plot of SRL under CK and the *Zm00001d051083* corresponding to the allelic effect of significant SNP; (**E**,**F**) Manhattan plot of SRSA under CK and the *Zm00001d051083* corresponding to the allelic effect of significant SNP; (**G**,**H**) Manhattan plot of PH under LN and the *Zm00001d025554* corresponding to the allelic effect of significant SNP; (**I**,**J**) Manhattan plot of TRL under CK and the *Zm00001d050798* corresponding to the allelic effect of significant SNP. In the figure, the upper edge of the boxplot represents the maximum value, the lower edge represents the minimum value, the open circles represent the mean, the filled circles represent the outliers, and contains a line representing the median. a and b indicate significance between CK and LN (*p* < 0.05).

Combining the GWAS results and the gene function annotations obtained by GO analysis, a total of four important candidate genes were identified. Under LN conditions, the SNP AX-86261031, which was significantly associated with CRN, was found on chromosome 2. The average CRN of the A allele at this locus was 5.43, which was significantly higher than 4 of the G allele. The candidate gene *Zm00001d004123* was involved in encoding lactoylglutathione lyase (Figure 5A,B). On chromosome 4, the SNP AX-86291150 was significantly associated with SRL and SRSA under CK conditions (Figure 5C,E). The average SRL and SRSA of the alleles at this locus were significantly different. The G allele was significantly better than the T allele (Figure 5D,F). The candidate gene *Zm00001d051083* may be the encoding gene of the LRR receptor-like serine/threonine protein kinase. Under LN conditions, SNP AX-86280646, which was significantly associated with PH, was found on chromosome 10. The average PH of the G allele was 19.8% higher than that of the A allele (Figure 5G,H). The candidate gene *Zm00001d025554* was related to the synthesis of aspartic protease (nepenthelin-2). Under CK conditions, the SNP AX-86313284, which was significantly associated with TRL, was found on the chromosome 4. The average TRL of the T allele was 309.15 cm, while the average TRL of the G allele was 246.46 cm (Figure 5I,J). The candidate gene *Zm00001d050798* was involved in the synthesis of E3 ubiquitin protein ligase EL5.

### 3.5. Candidate Gene Expression Results

**Figure 6:**
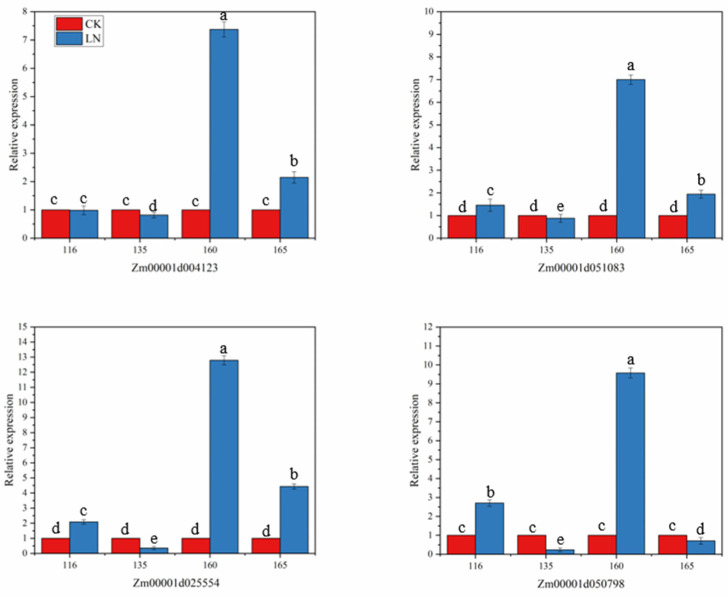
Expression analysis of four genes in roots of different sensitive lines of maize under nitrogen stress. In that order: The relative expression levels of genes *Zm00001d004123*, *Zm00001d051083*, *Zm00001d025554*, *Zm00001d050798* in roots of inbred lines 116, 135, 160 and 165 under low nitrogen stress. The inbred lines 116, 135, 160 and 165 represent four different haplotypes of inbred materials, of which 116 and 135 are unfavorable haplotypes, and 160 and 165 are favorable haplotypes. Error bars in the figure represent standard errors (*p* < 0.05). Significant difference between groups in each nitrogen level is indicated by different letters (*p* < 0.05).

When plants are under stress, they can inhibit or promote the synthesis or metabolism of some substances, thereby affecting the growth and development of plants. In order to understand the relationship between the four selected candidate genes and N stress, four maize lines with different N responses were selected for the differential expression analysis under N stress. As shown in Figure 6, the four candidate genes all responded to the LN stress, but the response levels were different. After being stressed, the expression of *Zm00001d004123* was slightly downregulated in the inbred line 135, there was almost no differential expression in inbred line 116, and it was upregulated in the other two inbred lines. Gene *Zm00001d25554* and *Zm00001d51083* were downregulated in the inbred line 135, and upregulated in the other three inbred lines, but the upregulation was smaller in the inbred line 165. The gene *Zm00001d050798* was downregulated in the inbred lines 135 and 165, especially in 135, which was significantly downregulated, and upregulated in 116 and 160. Under LN stress, the four candidate genes of the favorable haplotype inbred line 160 showed high upregulation, and the highest relative expression level reached 12.80. It can be seen that the inbred line 160 is more sensitive to LN. When the unfavorable haplotype inbred line 135 was under N stress, all four candidate genes were downregulated, and the lowest relative expression level was 0.24. The results of the candidate genes in response to LN stress indicated that these four genes were important genes related to LN tolerance and played an important role in the response to adversity stress.

## 4. Discussion

### 4.1. Phenotypic Evaluation of Traits Related to N Efficiency in Maize under Two N Levels

The N efficiency-related traits of maize mainly include morphological traits, yield traits, physiological and biochemical traits, etc. The 20 shoot and root traits in this study were all pointed out in previous studies to be related to N efficiency (Li et al., 2014; Yang et al., 2008; Chen et al., 2003) [18,19,20]. N efficiency-related traits were quantitative traits controlled by multiple environmental factors (Kant et al., 2011) [21]. There were obvious gene–environmental interaction effects. These factors cause N efficiency-related traits to be unstable, and they were extremely affected by other non-genetic factors, while being controlled by genes (Zhang et al., 1995) [22]. Previous studies (Li et al., 2015; Postma et al., 2014; Trachsel et al., 2013) [23,24,25] found differences in the nitrogen distribution between shoots and roots during the maize seeding stage under normal and low N supply conditions. LN conditions were more conducive to root growth. The specific performance was that the TRL, TRSA, and PRL were all higher than the normal N level. The suitable CRN and SRN were beneficial to N absorption under LN condition. In this study, the shoot traits (SPAD, PH, SDW) under normal N conditions were significantly higher than those in LN, and most root traits were significantly higher in LN conditions than in normal N application. The results of this study were consistent with previous studies. However, the TRSA, SRN and PRL were not significantly different under the two N levels. There was a difference from the previous research results, indicating that the response mechanism of maize to nitrogen stress was more complicated. Under different nitrogen levels, the maize root architecture changed, including the diameter and length of each root, the number of crown roots and the seminal roots, and the growth status of the lateral roots, so as to better capture N.

There was no significant correlation between SPAD and most of the traits under CK treatment. However, under LN conditions, SPAD was significantly positively correlated with PH, SDW and RDW, and significantly negatively correlated with RSR. It was also significantly positively correlated with the length and area of each part of the root system. This result indicated that the nutrient supply of each part of the plant was different under different nitrogen treatments. Plants preferentially satisfy the growth of the roots, and the growth status of the shoots was more obviously affected under LN stress (Li et al., 2021; Song et al., 2016) [26,27].

### 4.2. The Influence of Population Structure and Kinship on Association Analysis

The population structure plays an important role in carrying out the association analysis between traits and markers, which can reduce the false associations (Thornsberry et al., 2001) [28]. In this study, 124 maize inbred lines were divided into five subpopulations, according to the division basis of predecessors (Liu et al., 2009) [29]. However, the population structure used for the analysis increased the LD between SNPs on different chromosomes, and the association between the target traits and irrelevant sites caused the occurrence of false positives (Wu, 2009; Gaut and Long, 2003) [30,31]. It was necessary to consider the influence of the population structure on the association analysis. As an important factor affecting association analysis, kinship can theoretically lead to LD between non-linked markers, and the probability of LD between linked markers and non-linked markers was almost the same in maize (Wu, 2009; Stich et al., 2005) [30,32]. Therefore, an appropriate analysis method was essential for association analysis. This study adopted the GAPIT analysis method, including the five analysis models “GLM”, “MLM”, “CMLM”, “Farm CPU” and “Blink”. This method takes into account the influence of the population structure and kinship on association analysis. It can choose the statistical model best suited for the population and traits used in this study (Yang et al., 2011; Zhao et al., 2018) [33,34], which significantly improved the statistical power and calculation speed.

### 4.3. Genome-Wide Association Studies for Different Traits under Two N Levels

In previous studies (Li et al., 2016; Liu et al., 2011; Luo et al., 2015) [35,36,37], SNPs associated to N efficiency-related traits were distributed on 10 chromosomes of maize. In addition, Gallais and Hirel (2004) [38] and He (2018) [8] found that it was often difficult to locate the same marker for the same trait at different N levels. The expression of genes that control N efficiency changed under different N conditions. Some genes that were expressed under normal N conditions were inhibited under LN conditions. Other genes were not expressed in the presence of a sufficient N supply, but could be activated under LN conditions. In this study, the GWAS method was used to identify SNPs associated withN efficiency-related traits. As a result, 185 SNPs were detected, which were distributed on 10 chromosomes. For the same trait, most significant SNPs were not consistent under the two N levels, with the exception of AX-86273682, AX-86266372, AX-86250601, AX-86269378, AX-116873220 and AX-86295573 which were significantly associated with TLRL at the two N levels at the same time. In past research, many QTLs affecting the root phenotype were identified (Tuberosa et al., 2002; Zhu et al., 2006; Cai et al., 2012; Song et al., 2016; Liu et al., 2017) [27,39,40,41,42], however, it was difficult to identify consistent QTLs in different genetic populations. This study and Sun et al. (2020) [9] used different genetic populations to study in a similar growth environment, and the SNPs for the same traits were different, and no SNP consistent with other studies was found.

Tuberosa et al. (2002) [39] mapped QTLs for root-related traits at the seedling stage of maize under hydroponic conditions, and simultaneously mapped QTLs for yield and drought resistance coefficients under field drought and normal water conditions. A total of seven chromosomal regions could simultaneously detect QTLs for root traits and yield traits. Bertin and Gallais (2001) [43] used 99 RIL populations as research materials and carried out detailed QTL mapping for nitrogen efficiency-related traits through a two-year field experiment. Hirel et al. (2001) [44] used the same population to conduct hydroponic experiments, and the results of QTL mapping also had a good linkage relationship with the previous results. He (2018) [8] performed GWAS of nitrogen efficiency-related traits under field conditions using inbred populations, the chromosomal regions bin4.05~bin4.06 that control the nitrogen concentration in grains and the chromosomal region bin2.06 that control the nitrogen harvest index were detected. This study employed part of the He (2018) [8] genetic population for key loci mining of nitrogen efficiency-related traits under hydroponic conditions. Eleven significant loci controlling TRL, RDW, SRL, SRSA and CRV were detected in the chromosomal region bin4.05~bin4.06, and nine significant loci controlling CRN, PH, CRN, PRL and PRSA were detected in the chromosomal region bin2.06. From these results, it can be inferred that the research on the significant loci at the seedling stage of maize under hydroponics has the same characteristics as the real physiological conditions of the field environment, so the research at the seedling stage is also of great significance.

### 4.4. Complex Molecular Mechanisms of Root and Shoot Growth under Different N Conditions

Based on the results of GWAS and the annotated genes of SNPs within 200 kb on the B73 genome, four candidate genes were identified in the regions with significant SNP and combined with the results of candidate gene expression analysis, the possible molecular mechanisms affecting the growth of maize seedlings stage were explored. (1) The gene *Zm00001d004123* was located on chromosome 2 and contains SNP that was significantly associated with CRN. This gene was involved in the synthesis of lactoylglutathione lyase (glyoxalase I), which was involved in the detoxification system of methylglyoxal (Maurino and Engqvist, 2015; Bhowal et al., 2020) [45,46], and salicylic acid (Mostofa and Fujita, 2013) [47], brassinosteroids (Alam et al., 2019; Jan et al., 2020) [48,49], cytokinin (Nishiyama et al., 2012) [50] and other plant hormones, can help improve plant stress tolerance (Singla-Pareek et al., 2003) [51]. Nitrate played an important role in regulating the growth of lateral roots for plants (Gan et al., 2012) [52]. Glyoxalase may maintain appropriate lateral root growth and improve the ability of N stress by regulating the physiological activities. Studies have shown (El-Shabrawi et al., 2010) [53] that under salt stress, salt-tolerant varieties have higher glyoxalase activity than salt-sensitive varieties in rice seedlings. In this study, low-nitrogen tolerant materials not only promoted lateral root growth, but also upregulated the expression of gene *Zm00001d004123* under LN treatment. However, the expression of these genes was not significantly upregulated or even downregulated when the sensitive material was stressed by LN. It can be proved that the upregulation of gene *Zm00001d004123* can increase the glyoxalase activity in plants under LN, and promote lateral root growth, which can improve the tolerance of maize; (2) The candidate gene *Zm00001d051083* contains a SNP that was significantly associated with SRL and SRSA. It was located on chromosome 4 and participates in the encoding of LRR receptor-like serine/threonine protein kinase. Jia et al. (2020) [54] found that a gene *KNR6* encoding serine/threonine protein kinase can affect the ear length and grain number in maize. *GsSRK* had a highly conserved serine/threonine protein kinase catalytic region. Sun et al. (2013) [55] found that the overexpression of *GsSRK* in Arabidopsis promoted seed germination and root growth, and that this gene was induced by stress conditions. Azad and Alemzadeh (2017) [56] showed that when maize was subjected to phosphate stress, the expression of the *ZmSTPK1*, which encodes serine/threonine protein kinase, responded to effective phosphate, and its expression was upregulated in the stressed plants. Similar to the results of previous studies, when maize is under nitrogen stress, the expression of gene *Zm00001d051083* is mainly upregulated. The difference was that expression in one of the nitrogen-sensitive varieties was slightly downregulated, which may be related to genetic differences between the lines; (3) The candidate gene *Zm00001d025554* was located on chromosome 10 and contains a SNP significantly associated with PH. This gene encodes an aspartic protease (AP). Xia et al. (2004) [57] pointed out that the overexpression of the aspartic protease-encoding gene in Arabidopsis leads to plant dwarfing. Wang et al. (2020) [58] cloned dwarf-related genes from dwarf castor-oil plants, and over-expressed them to obtain the purified target protein, aspartic protease. Under drought treatment, the expression of the AP gene *ASPG1* in Arabidopsis thaliana was upregulated, which positively regulated abscisic acid synthesis genes and enhanced the ability of plants to resist drought. Overexpression of *CDR1*, the gene encoding AP of Arabidopsis thaliana, enhanced plant resistance to disease and showed a dwarf phenotype (Wang and Wu, 2016) [59]. Compared with previous research results, *Zm00001d025554* was located at PH, and PH decreases under LN stress, gene expression was upregulated and plant stress resistance was regulated by gene expression; (4) *Zm00001d050798* was located on chromosome 4, contains a SNP significantly associated with TRL, and participates in the synthesis of E3 ubiquitin protein ligase EL5. In the study of Mochizuki et al. (2014) [60], the EL5 protein was involved in the maintenance of root meristematic viability in rice. Cytokinin and superoxide were produced in the nitrogen supply pathway, which led to changes in the root system. EL5 preferentially acts on root development by maintaining the proliferation and viability of primordial cells in the crown roots and the lateral roots. Increased sensitivity to nitrogen in EL5-overexpressing rice plants, compared with normal plants, the expression of nitrite-responsive genes was upregulated. After being treated with nitrite, it will cause the death of the root tip meristem cells and inhibit root development. Therefore, the LN environment was more conducive to plant root development. So, not only can EL5 be expressed normally and maintain root cell viability, it can also avoid the sensitive response to nitrogen after EL5 expression is upregulated.

Luo (2020) [61] screened seven candidate genes using GWAS under the condition of salt treatment at the seedling stage. Among them, the germination rate of arabidopsis thaliana materials overexpressing *ZmSAG4* was significantly higher than that of the wild type, which proved that the candidate gene is a positive regulator of plant salt stress. According to the SNP of the *ZmSAG4*, the materials of different haplotypes were screened, and the extremely salt-tolerant and extremely sensitive inbred lines were used as materials for salt treatment. The results showed that the plant height, fresh weight and biomass of the highly resistant materials were significantly larger than those of the sensitive materials. In this research, according to the expression results of important candidate genes of four maize inbred lines, it was found that the candidate genes related to nitrogen efficiency are upregulated in nitrogen-efficient inbred lines, and the expression level was significantly higher than that of the nitrogen-inefficient inbred lines. The four candidate genes were all upregulated in the nitrogen-efficient inbred line 160 and downregulated in the nitrogen-inefficient inbred line 135. Combined with the analysis of the field phenotype and yield results (unpublished), the nitrogen-efficient inbred line 160 showed high tolerance to low nitrogen, and showed significant advantages in phenotype, dry matter accumulation and yield from the silking stage to maturity stage. However, the nitrogen-inefficient inbred line 135, with downregulated expression of the candidate gene, showed poor growth. It can be speculated that the nitrogen-efficient inbred lines can improve the low nitrogen tolerance and maintain a relatively good growth condition by regulating the expression of nitrogen efficiency-related genes under nitrogen stress conditions.

## 5. Conclusions

Nitrogen efficiency-related traits in maize are quantitative traits affected by genes and many other non-genetic factors. In the present study, the growth status of nitrogen-related traits in the maize seedling stage under different nitrogen levels was observed, and genome-wide association studies were performed. According to the measurement results, the root morphological construction changed with the nitrogen content in the environment. In the absence of nitrogen, the root nutrients were preferentially supplied. According to the results of the GWAS analysis, 185 SNPs distributed over 10 chromosomes were detected among them, of which six SNPs significantly affected the TLRL at the two nitrogen levels. Based on the GWAS results and the annotated genes obtained from previous studies, four candidate genes that significantly affect the traits related to nitrogen efficiency were screened out. Candidate genes all respond to the LN environment, and their expression is upregulated in favorable haplotype inbred lines, but there are differences in expression in unfavorable haplotype inbred lines. Based on these results, some important information was provided for exploring the physiological and possible molecular mechanisms of maize seedling growth.

## Figures and Tables

**Table 1:** Maize seedling trait measurements and descriptions.

Trait Name	Abbreviations	Trait Measurements and Descriptions
SPAD value of the 2nd leaf	SPAD	Measured with a chlorophyll meter
Plant height (cm)	PH	Measured with a ruler
Shoot dry weight (g)	SDW	Dried and weighed on a balance (0.001 g)
Root dry weight (g)	RDW	Dried and weighed on a balance (0.001 g)
Root-to-shoot ratio	RSR	Root dry weight/shoot dry weight
Total root length (cm)	TRL	Length of the whole root system
Total root surface area (cm^2^)	TRSA	Surface area of the whole root system
Total root volume (cm^3^)	TRV	Volume of the whole root system
Crown root number	CRN	Count of the axial first whorl crown roots
Crown root length (cm)	CRL	Measured with a ruler
Crown root surface area (cm^2^)	CRSA	Measured with root scanner
Crown root volume (cm^3^)	CRV	Measured with root scanner
Seminal root number	SRN	Count of the axial seminal roots
Seminal root length (cm)	SRL	Measured with a ruler
Seminal root surface area (cm^2^)	SRSA	Measured with root scanner
Seminal root volume (cm^3^)	SRV	Measured with root scanner
Primary root length (cm)	PRL	Measured with a ruler
Primary root surface area (cm^2^)	PRSA	Surface area of the whole primary root
Primary root volume (cm^3^)	PRV	Volume of the whole primary root
Total lateral root length	TLRL	The total length of all of the lateral roots of the entire root system

**Table 2:** Number of significant SNPs detected for each trait.

Traits	Env		Total
	CK	LN	
SPAD	7	2	9
PH	9	2	11
SDW	6	1	7
RDW	2	4	6
RSR	0	9	9
TRL	1	7	8
TRSA	1	2	3
TRV	2	1	3
CRN	14	1	15
CRL	3	6	9
CRSA	3	2	5
CRV	6	3	9
SRN	3	1	4
SRL	2	4	6
SRSA	4	3	7
SRV	1	4	5
PRL	10	0	10
PRSA	5	3	8
PRV	11	13	24
TLRL	11	16	27
Total	101	84	185

## Data Availability

Not applicable.

## Supplementary Materials

The following supporting information can be downloaded at: https://www.mdpi.com/article/10.3390/plants11111417/s1.
