# Supplementary material for: Genome-Wide Association Studies of Maize Seedling Root Traits under Different Nitrogen Levels
Source: Plants (Basel). 2022 May 26;11(11):1417. doi: 10.3390/plants11111417 (PMC9182862; doi:10.3390/plants11111417)
Supplement: Supplementary file 1 [file plants-11-01417-s001.zip › Figure S1.pdf]

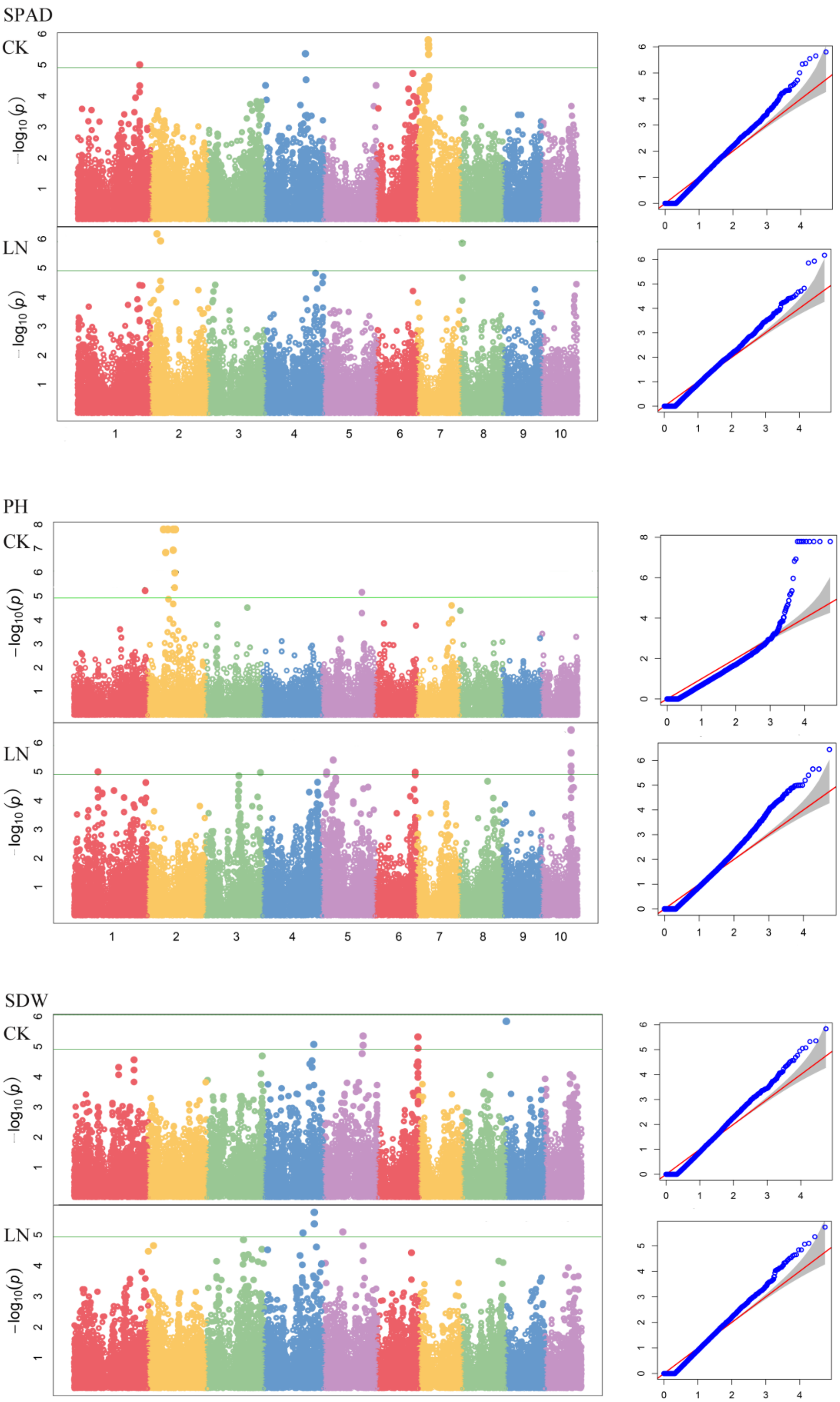
SPAD
CK
LN
PH
CK
LN
SDW
CK
LN
−log10(p)
1
2
3
4
5
6
7
8
9
10

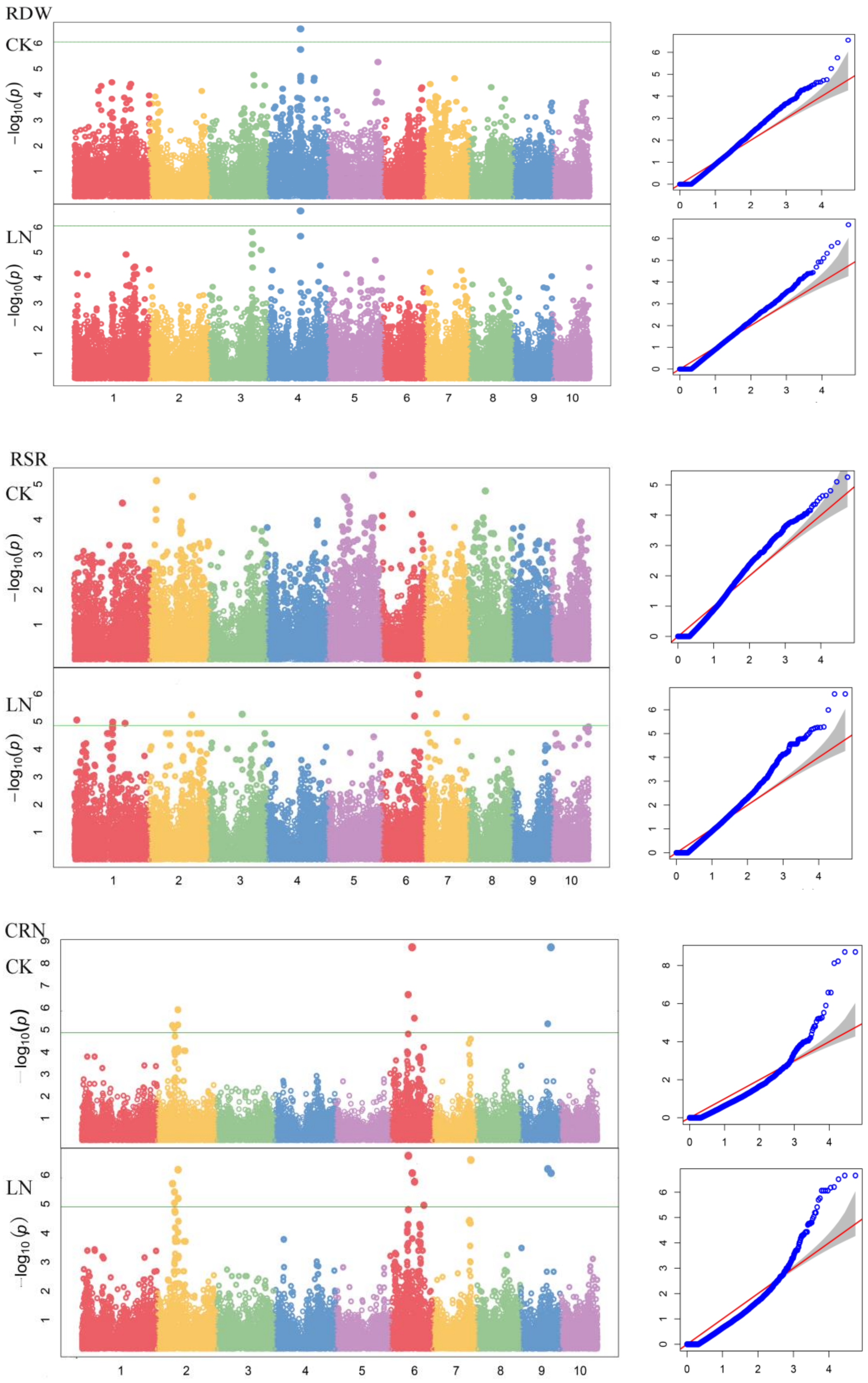
RDW
CK
LN
RSR
CK
LN
CRN
CK
LN
−log10(p)
1
2
3
4
5
6
7
8
9
10

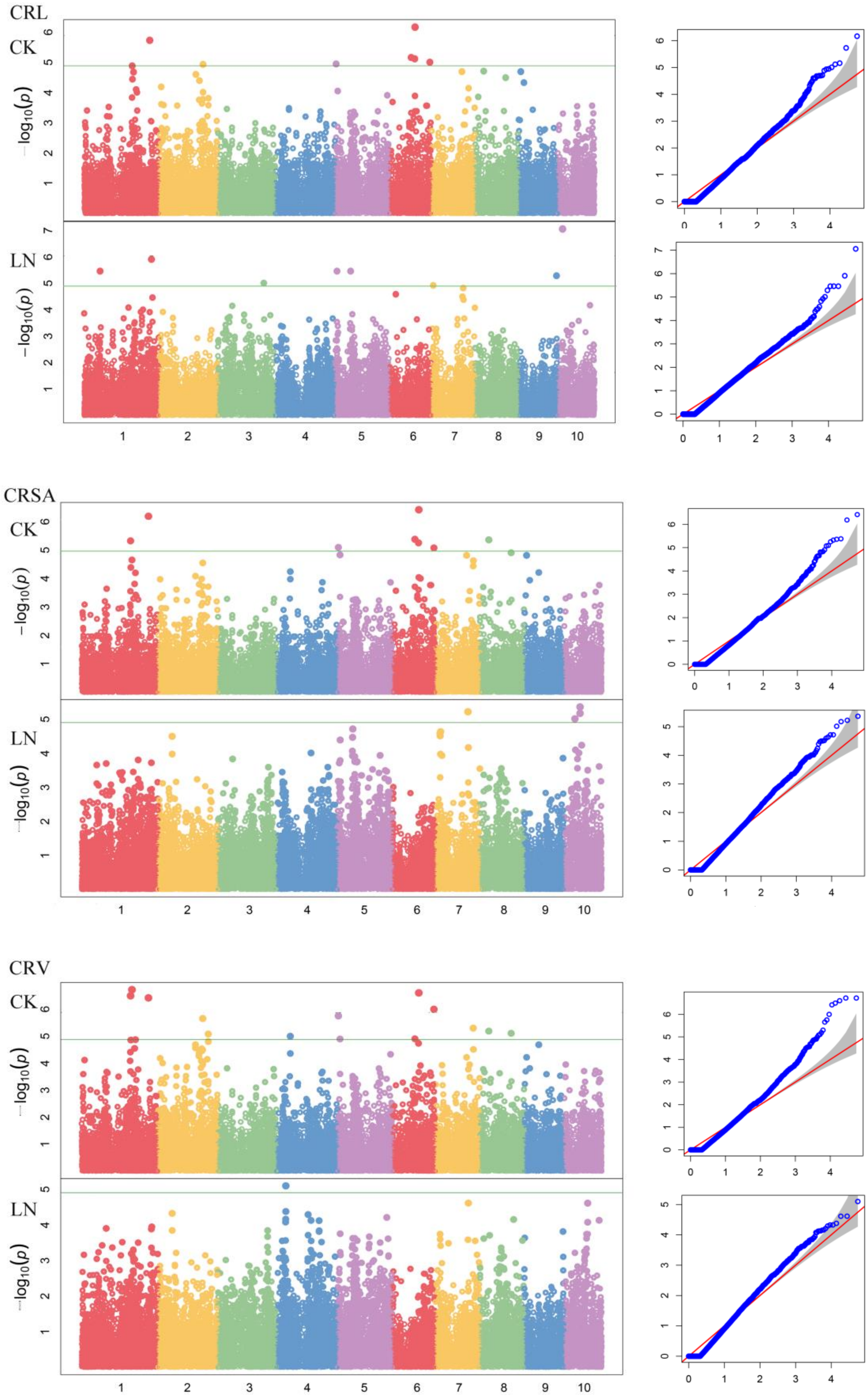
CRL
CK
LN
CRSA
CK
LN
CRV
CK
LN
$-\log_{10}(p)$
1
2
3
4
5
6
7
8
9
10

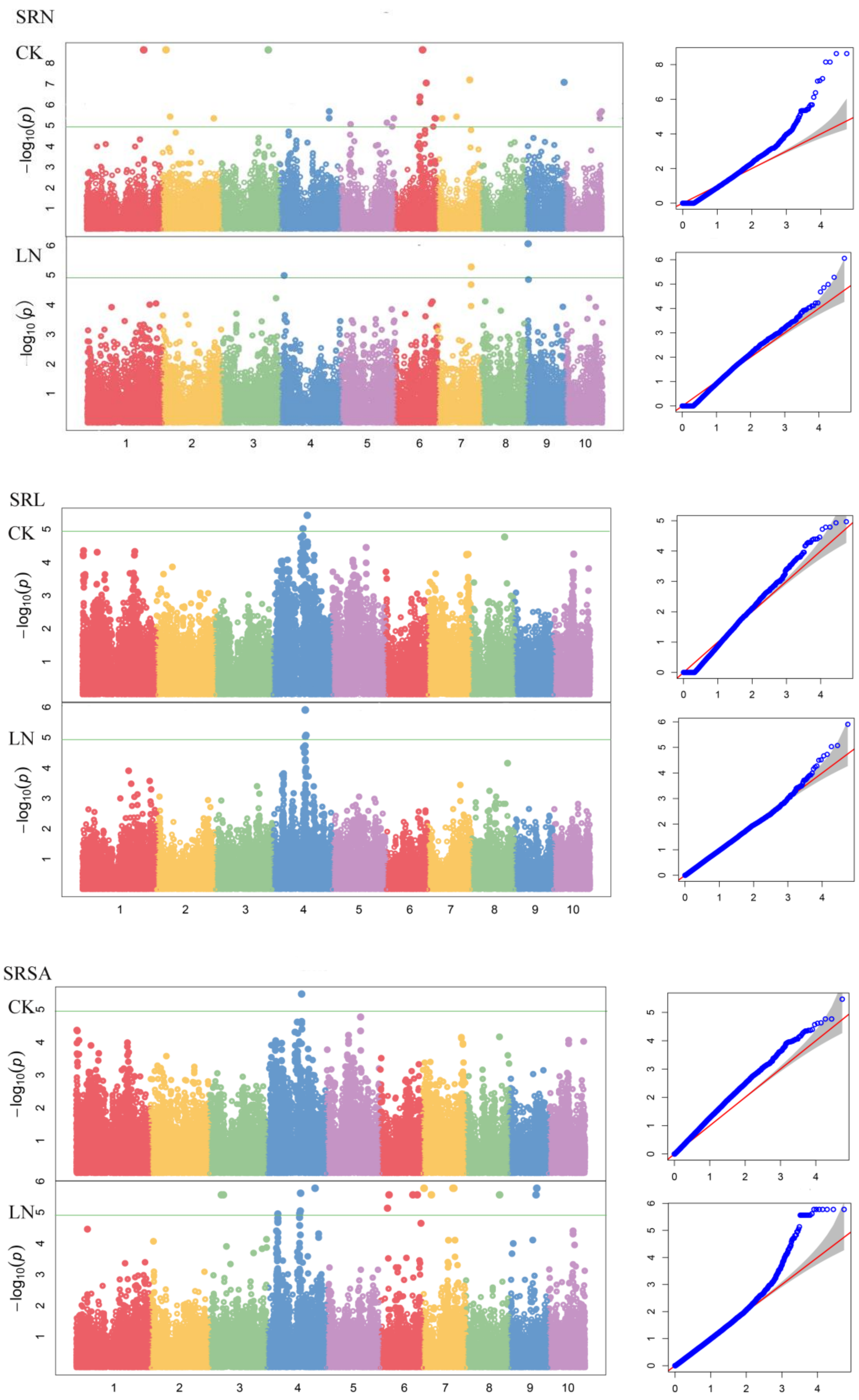
SRN
CK
LN
SRL
CK
LN
SRSA
CK
LN
$-\log_{10}(p)$
1 2 3 4 5 6 7 8 9 10

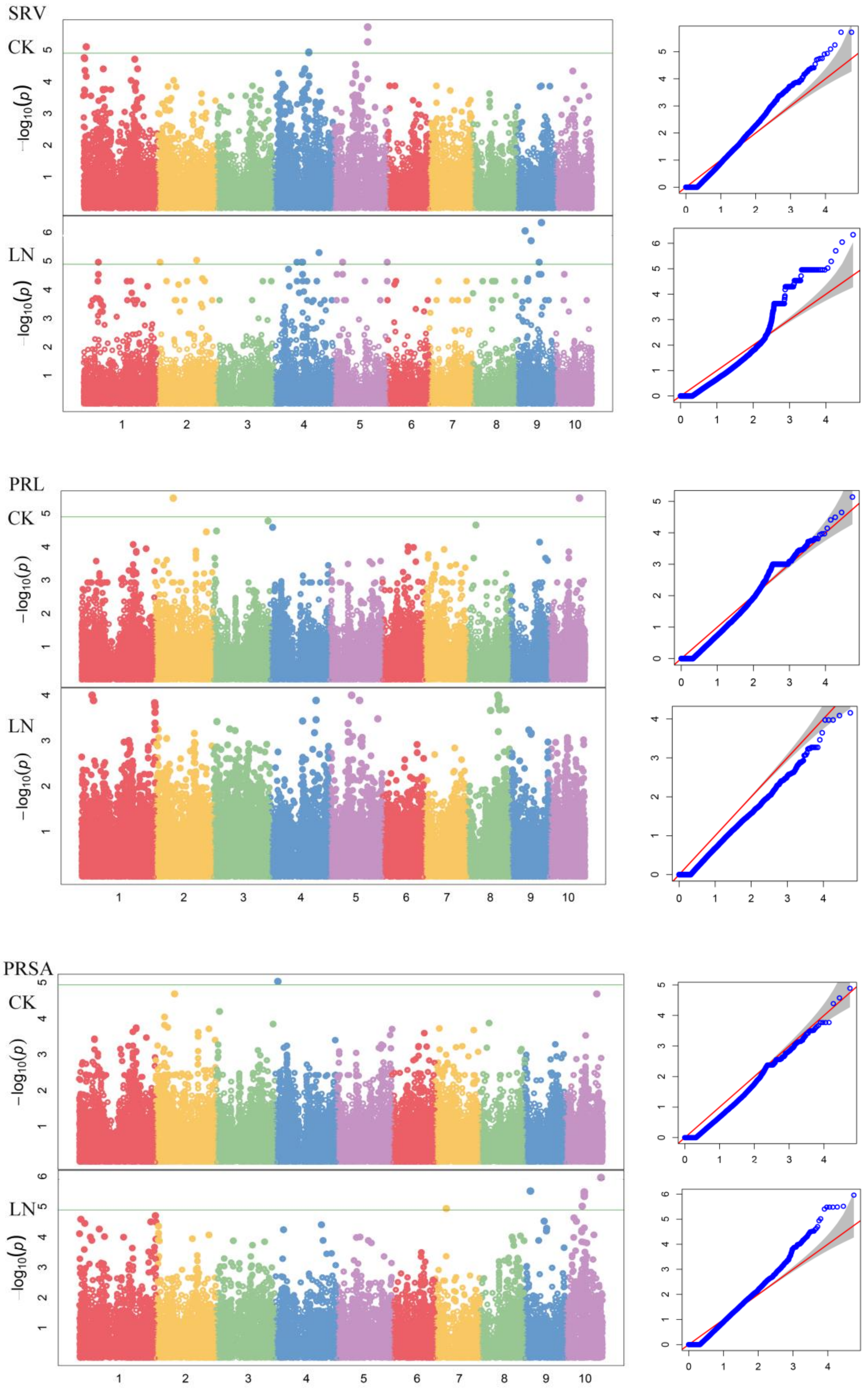
SRV
CK
LN
PRL
CK
LN
PRSA
CK
LN
−log10(p)
1 2 3 4 5 6 7 8 9 10

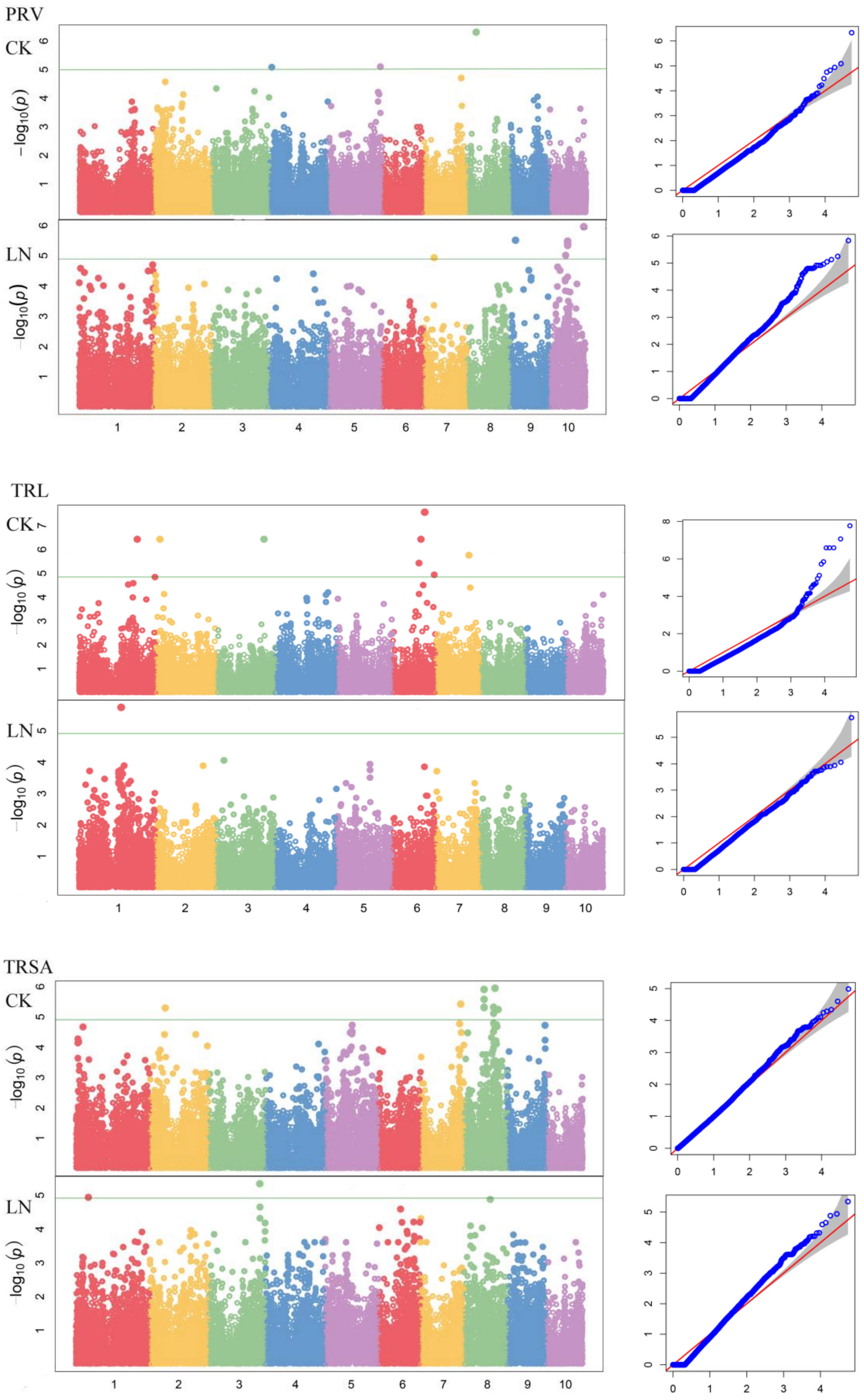
PRV
CK
LN
TRL
CK
LN
TRSA
CK
LN
$-\log_{10}(p)$
1 2 3 4 5 6 7 8 9 10

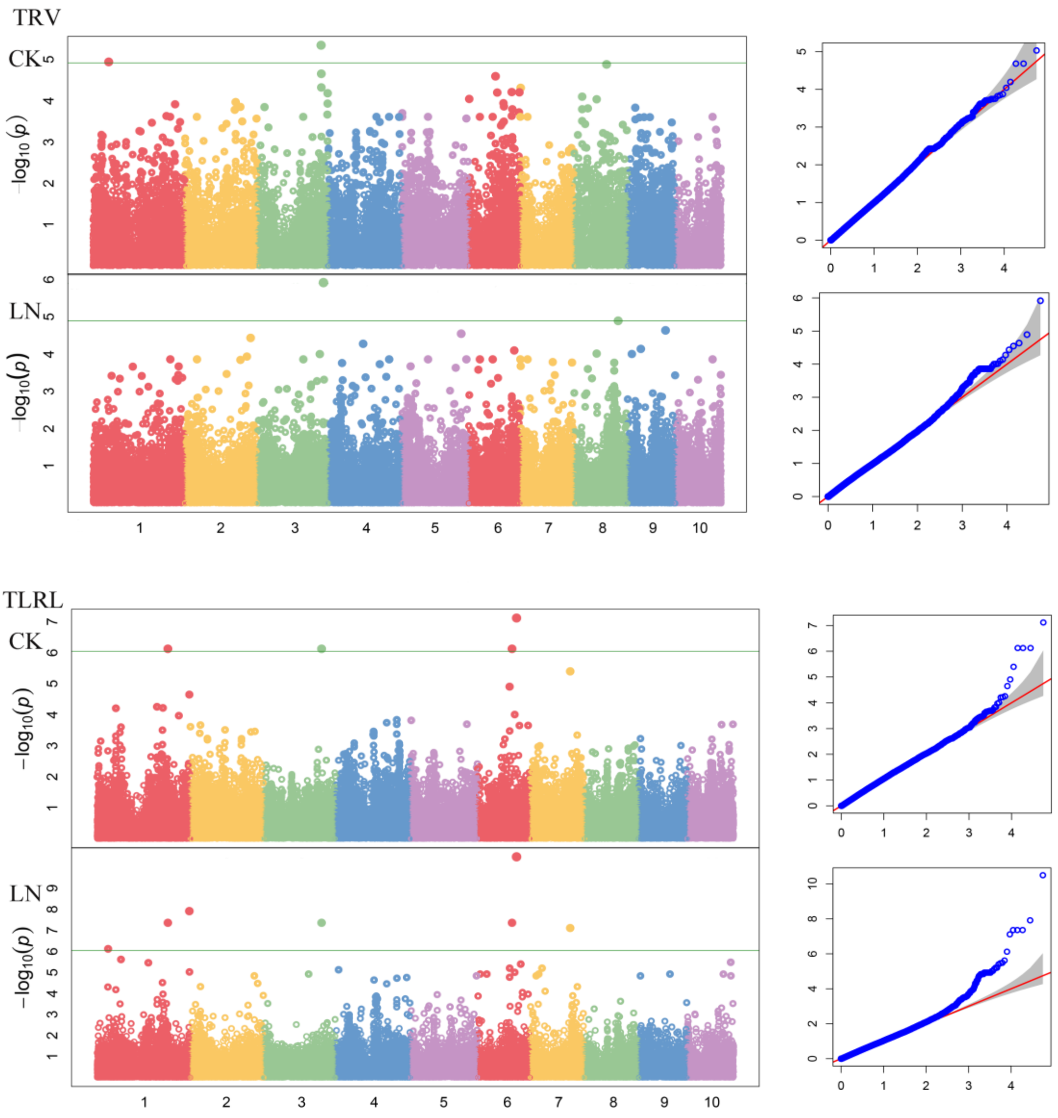

Figure S1. Manhattan plot and QQ plot of 20 traits under CK and LN The horizontal line in the figure indicates the significant −log10 (P) value.
