# Supplementary figures and images for: Genome-Wide Association Studies of Maize Seedling Root Traits under Different Nitrogen Levels
Source: Plants (Basel). 2022 May 26;11(11):1417. doi: 10.3390/plants11111417 (PMC9182862; doi:10.3390/plants11111417)

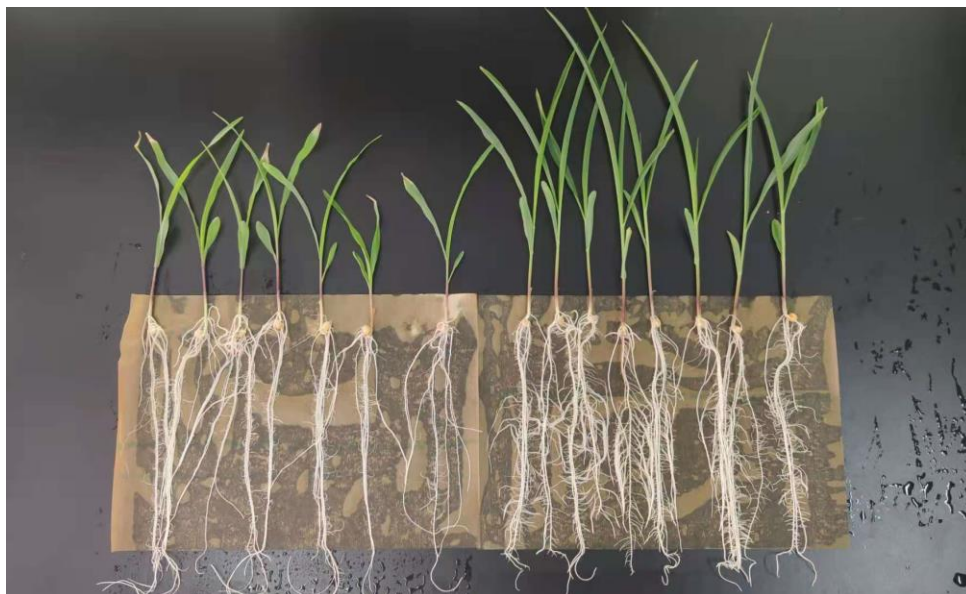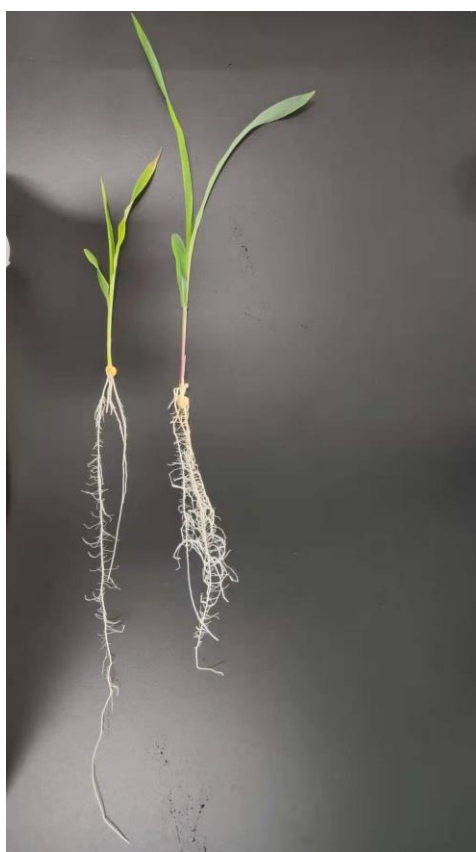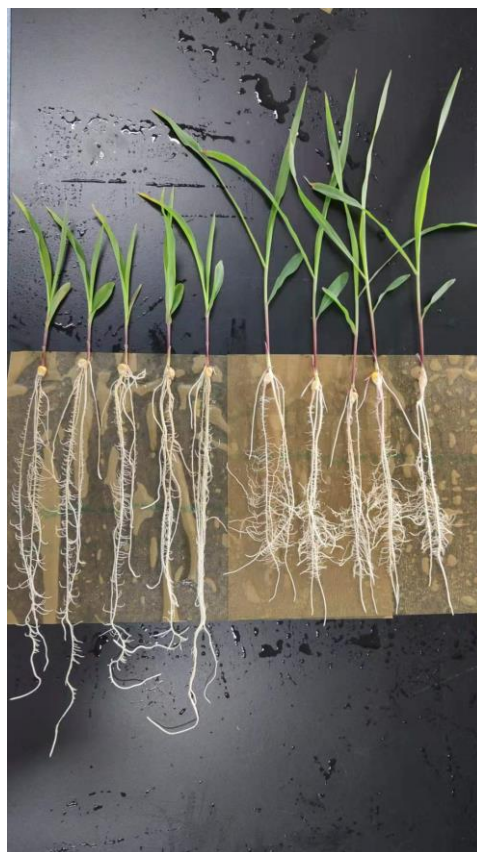

**Figure S7 Phenotypic Results Plot**

Supplement: Supplementary file 1 [file plants-11-01417-s001.zip › Figure S7.pdf]
